# Patterns of geographic variation of thermal adapted candidate genes in *Drosophila subobscura* sex chromosome arrangements

**DOI:** 10.1186/s12862-018-1178-1

**Published:** 2018-04-24

**Authors:** Pedro Simões, Marta Pascual

**Affiliations:** 1 Departament de Genètica, Microbiologia i Estadística and IRBio, Facultat de Biologia, Universitat de Barcelona, 08028 Barcelona, Spain; 2 Present Address: cE3c - Centre for Ecology, Evolution and Environmental Changes, Faculdade de Ciências, Universidade de Lisboa, Campo Grande, 1749-016 Lisbon, Portugal

**Keywords:** Chromosomal arrangements, *Drosophila subobscura*, UTR variation, Genetic differentiation, Gene flow, Geographic variation, Clinal variation, Climatic selection, Thermal adaptation

## Abstract

**Background:**

The role of chromosomal arrangements in adaptation is supported by the repeatable clinal variation in inversion frequencies across continents in colonizing species such as *Drosophila subobscura.* However, there is a lack of knowledge on the genetic variation in genes within inversions, possibly targets of climatic selection, across a geographic latitudinal gradient. In the present study we analysed four candidate loci for thermal adaptation, located close to the breakpoints, in two chromosomal arrangements of the sex (A) chromosome of *Drosophila subobscura* with different thermal preferences*.* Individual chromosomes with A_2_ (the inverted arrangement considered warm adapted) or A_ST_ (the standard ancestral arrangement considered cold adapted) were sequenced across four European localities at varying latitudes, up to ~ 2500 Kms apart.

**Results:**

Importantly, we found very low differentiation for each specific arrangement across populations as well as no clinal patterns of genomic variation. This suggests wide gene exchange along the cline. Differentiation between the sex chromosome arrangements was significant in the two more proximal regions relative to the A_ST_ orientation but not in the distal ones, independently of their location inside or outside the inversion. This can be possibly due to variation in the levels of gene flux and/or selection acting in these regions.

**Conclusions:**

Gene flow appears to have homogenized the genetic content within-arrangement at a wide geographical scale, despite the expected diverse selective pressures in the specific natural environments of the different populations sampled. It is thus likely that the inversion frequency clines in this species are being maintained by local adaptation in face of gene flow. The differences between arrangements at non-coding regions might be associated with the previously observed differential gene expression in different thermal regimes. Higher resolution genomic scans for individual chromosomal arrangements performed over a large environmental gradient are needed to find the targets of selection and further elucidate the adaptive mechanisms maintaining chromosomal inversion polymorphisms.

**Electronic supplementary material:**

The online version of this article (10.1186/s12862-018-1178-1) contains supplementary material, which is available to authorized users.

## Background

There is increasing evidence of the role of chromosomal inversion polymorphisms in adaptation and speciation [1, 2]. Several lines of evidence attest this namely: (1) the repeated occurrence of latitudinal clinal variation across multiple invaded continents, particularly in Drosophila species ([3–6]; (2) consistent associations between inversions and several relevant phenotypic traits [7–13]; and (3) inversion frequency changes beyond genetic drift expectations across replicated populations adapting to imposed laboratory conditions [14–16].

Several hypotheses have been advanced to explain the adaptive character of inversions (see [1]), with the most widely accepted hypotheses relying on recombination reduction in inversion heterokaryotypes and consequent protection of genes with positive effects on fitness [17, 18]. The coadaptation hypothesis [17] postulates that specific favourable combinations of alleles interacting epistatically within a particular inversion arise as an adaptive response to a given environment. The local adaptation hypothesis [18] states that the spread of a new inversion results from advantageous locally adapted alleles captured within the inverted segment, without epistasis being necessarily involved. Inversion frequencies changes will result from a balance between selection and migration. A general expectation in both these scenarios is that there is genetic differentiation between inversion types, as a result of local adaptation shaping the genetic content of different inversions. Coalescent models suggest that this differentiation is likely higher near breakpoints – where recombination is greatly reduced - and near locally adapted alleles within the inversion [19]. In addition, under the coadaptation hypothesis there is a clear expectation of genetic differentiation within inversions sampled from different geographical populations, as a result of population-specific epistatic interactions evolving in response to different environmental conditions [20]. However, this pattern might not be exclusive of the co-adaptation model as some within-inversion differentiation might occur depending on the balance between migration and selection on locally adapted alleles (with or without epistasis involved) as postulated by the local adaptation.

Empirical studies on the molecular variation associated with inversions have frequently detected high levels of genetic differentiation between chromosomal arrangements ([21–27] but see [28]). This pattern is consistent with the aforementioned hypotheses and is expected as a result of reduced recombination in inversion heterokaryotypes leading to distinct genetic pools. Studies in the O chromosome of *D. subobscura* found an extensive genetic differentiation between arrangements occurring regardless of the distance to the nearest breakpoint, with selection likely implicated [29, 30]. On the other hand, several studies have reported a general lack of measurable genetic differentiation within arrangements sampled from different populations ([20, 31–33], contrary to the expectations of the coadaptation hypothesis. An important exception is the genetic clinal variation within the inversion *In(3R)Payne* of *Drosophila melanogaster*, both in North American and Australian localities, implicating a role of this inversion in the response to climatic selection [6, 31, 34]. In addition, there is some evidence for epistasis - as expected under the coadaptation model - based on patterns of linkage disequilibrium (LD) with regions within arrangements showing high levels of LD and differentiation interspersed with other regions of low LD [20, 31] as well as LD between arrangements and genes located outside them [30].

One of the most emblematic examples of the adaptive role of chromosomal polymorphisms comes from *Drosophila subobscura*, a species presenting repeatable clinal patterns for inversion frequencies across Europe, the ancestral area, and North and South America [3]. Thermal adaptation or associated factors might be implicated in such patterns. Evidence for this hypothesis comes from the association between temporal frequency changes of *D. subobscura* chromosomal arrangements and increased temperature due to climate warming at a worldwide scale [5]. Also, seasonal inversion frequency changes in *D. subobscura* have been reported, even shortly responding to heat waves [35] as well as associations between inversions and several phenotypic traits, including thermal preference and tolerance [36–38] and body size [10, 39]. Importantly, differences in gene expression patterns were also observed in *D. subobscura* from a colonizing South American population evolving in the laboratory at different thermal regimes (13, 18 and 22 °C); with an enrichment for candidate genes located inside arrangements [40]. This suggests that different inversions may contribute to the maintenance of genes with differential expression and potential impact on fitness. Indeed, this was the case in *D. pseudoobscura* with multiple genes presenting expression differences among arrangements [41]. Consequently, it is relevant to analyse overall patterns of molecular variation in these candidate genes in the vicinity/within the *D. subobscura* inversions as well as address their variation across the species geographic gradient to search for targets of climatic selection.

Pegueroles et al. [33] analysed 6 of these genes previously associated with thermal adaptation located in the O chromosome of *D. subobscura* and found no differentiation for the same arrangement between Barcelona and Greece indicating high levels of gene flow and the absence of geographic varying selection at least in those two southern regions. In a study in *D. subobscura* involving several localities across western Europe, Simões et al. [32] using microsatellite loci also detected low within-arrangement differentiation in several inversions of two autosomes (J and U) and the sex chromosome (A) confirming an extensive gene flow in this species (see also [42, 43]). Nevertheless, significant clinal patterns at few specific microsatellite loci within arrangements emerged (one in the distal region of A_2_), suggesting adaptation at neighbouring loci.

The aim of the present study is to examine the genetic variation associated with two arrangements of the sexual chromosome of *Drosophila subobscura* across a geographic gradient. Specifically, we intend to (1) characterize genetic variation and differentiation between arrangements, addressing the impact of inverted regions at the genetic level; (2) analyse patterns of within-arrangement differentiation, searching for evidence of different epistatic combinations in different populations; (3) identify latitudinal patterns of genetic variation, which might indicate targets of climatic selection at a geographical scale. In this study we targeted A_ST_ and A_2_, the two most frequent inversions of the A chromosome in our sampled localities. These two arrangements present clinal variation of frequencies in Europe, as well as in the two recently colonized American hemispheres, with A_2_ increasing frequency towards warmer locations and A_ST_ showing the corresponding opposite pattern [32, 44]. We analysed DNA sequence variation in four gene regions (*PhKgamma*, *Ubc-E2H*, *Hfw* and *Nipsnap*). These genes are good candidates to search for evidences of climatic latitudinal selection associated with the *Drosophila subobscura* inversion clines as they (1) have been previously implicated in thermal adaptation ([40], see also above) and (2) are located relatively close to inversion breakpoints, with reduced recombination in these regions possibly protecting genetic variants and showing the footprint of selection.

## Methods

### Fly samples and chromosomal inversion scoring

**Fig. 1:**
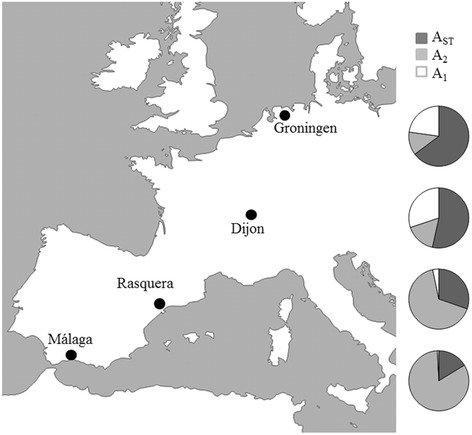
Sampling sites of *D. subobscura* and frequency, as coloured pies for each locality, of the three sex chromosome arrangements detected (data from [25]). A_ST_ – dark grey, A_2_ – light grey; A_1_ – white

Wild *Drosophila subobscura* samples were collected from four European locations (Fig. 1). Samples from Málaga (Mal, 36° 43’N, Spain) and Rasquera (Ras, 40° 57’N, Spain) were obtained in October 2008 with a total of 169 and 152 individuals, respectively. Samples from Dijon (Dij, 47° 18’N, France) and Groningen (Gro, 57° 13’N, Netherlands) were obtained in August/September 2009 with a total of 344 and 326 individuals, respectively [45]). These locations vary greatly in environmental conditions, for instance the two most extreme sampled locations have presented clearly contrasting range of yearly average temperatures (7–31 °C in Málaga vs. 0–23 °C in Groningen) and also average precipitation values (44 mm in Málaga vs. 75 mm in Groningen) between 2000 and 2012. We performed the collections in the late summer/early fall to capture the second peak of abundance for the species in the different locations allowing a more reliable comparisons between warmer and colder populations [46].

Our study complies with National and International guidelines, either involving field collections or experimentation in animals. We use *Drosophila subobscura,* which is not a threatened species nor requires legal ethical consent for experimentation.

**Fig. 2:**
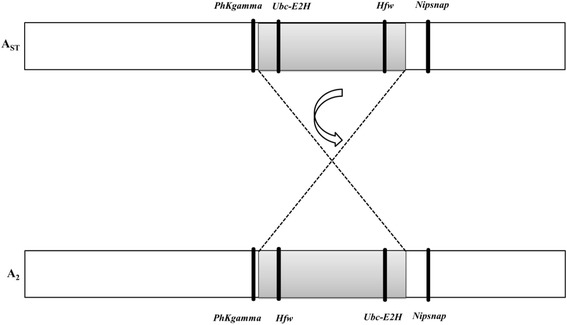
Cytological location of the genes analysed. The grey box represents the region inverted in the A_2_ arrangement. Black bars indicate the cytological location of each gene in each chromosomal arrangement

This study focused on the two most frequent arrangements present on the acrocentric A (sexual) chromosome (A_ST_/A_2_), and the only two arrangements present in all analysed locations (Fig. 1). A_ST_ is considered an ancestral chromosome arrangement and A_2_ a derived medium-sized inversion (~ 7.1 Mb) with an estimated age of 160.000 years [23]. The breakpoints of A_2_ (Fig. 2) are 8C/D-12C/D [47]. In order to score the chromosomal arrangements of the A chromosome, wild-caught males or F_1_ male descendants from wild isofemale lines were individually crossed with virgin females of the *chcu* strain, homokaryotypic for the A_ST_ arrangement. Polytene chromosomes from salivary glands of one female larva descendant per cross were stained with 2% orcein in 60% acetic/lactic acid (50:50) (see details in [32]). This protocol allowed identifying the chromosomal arrangement of each parental male from the chromosomal configuration of their female larva progeny.

### Genomic regions, DNA extraction and sequencing

DNA was individually extracted from wild males (or male descendants of wild females) whose chromosomal arrangements (A_2_ or A_ST_) had been previously scored as described above. By amplifying and directly sequencing males we avoided the problems associated with sequencing of diploid data. The karyotype of these males was inferred from their descendants (see above) making it possible to obtain both genetic and karyotypic data for the same individual. Genomic DNA extraction was performed following the protocol described in [48]. The four genomic regions analysed were localized again by in situ hybridization with digoxigenin following standard protocols [49], as some genes in Laayouni et al. [40] have proved to be mislocated [33]. All probes hybridized in the same band previously reported [40] with the exception of *Nipsnap* that was localized nearby but outside the inversion (see Fig. 2 and Additional file 1). Thus, *PhKgamma* (cytological location 8C; CG1830) and *Nipsnap* (13B; CG9212) are located outside the A_2_ inversion, and *Ubc-E2H* (9A; CG2257) and *Hfw* (12A; CG3095) within that inversion (Fig. 2). Following the A_ST_ arrangement, being the ancestral one, we will refer to *PhKgamma* and *Ubc-E2H* as the proximal loci, the region being closer to the centromere, and *Hfw* and *Nipsnap* as the distal loci. *Ubc-E2H* (Ubiquitin conjugating enzyme E2H) is involved in lateral inhibition and protein polyubiquitination, *PhKgamma* (Phosphorylase kynase enzyme) is involved in embryonic morphogenesis and glycogen metabolism and *Hfw* (Halfway) is involved in intracellular signal transduction and pupariation and no involvement in biological processes is yet ascribed to the *Nipsnap* gene although a putative role in vesicular transport has been proposed (the function of each gene is according to Flybase). Primers used for amplification and sequencing reactions are listed in Table 1. The fragments amplified correspond to partial sequences, not comprising the totality of each gene (see Table 1 for details). Distance to the nearest breakpoint (Mb) was estimated by counting the number of cytological bands in the map of Kunze-Mühl and Müller [50] assuming that all bands contain the same DNA content (82 Kb) as in [51]. Distances were around 0.082 Mb for *PhKGamma* and around 0.574 Mb for the other 3 genes (Fig. 2).

**Table 1:** Chromosomal location, length and primers used for each of the 4 genes studied

Gene^a^	Alignment^b^	Primers (Forward; Reverse)	5’UTR	Exons	Introns
*PhKgamma* (CG1830, GA14880)	831 bp (895 bp)	5′ - CATGACCTGGCGCAGTATTG - 3′ 5′ - TAACAGCGGAGCGAGCAGTC - 3’	1–240	241–332	333–895
*Ubc-E2H* (CG2257, GA15327)	1085 bp (1100 bp)	5’ - TCACTGTAGTCGGACATGCT - 3′ 5′ - AGGAGAGCAACGTCACAGAT - 3’	1–662	663–1100	–
*Hfw* (CG3095, GA15922)	1073 bp (1079 bp)	5’ - GCATTCAAGCGGTCCGTTAA - 3′ 5′ - TATGTTGAGGCACTTGAGCG - 3’		349–1079	1–348
*Nipsnap* (CG9212, GA21617)	1095 bp (1168 bp)	5’ - CATGCGACTGTGAGCCTCTT - 3′ 5′ -CGCAGCAATACAACAAGTGG - 3’	1–411	412–470; 1149–1168	471–1148

^a^Gene symbol of the homologous gene in *D. melanogaster* and *D. pseudoobscura* respectively in parentheses

^b^alignment length with *D.pseudoobcura* in parentheses. 5’ UTR, introns and exon positions are referred relative to the *D. pseudoobscura* alignment

DNA amplification and sequencing was carried out as in [45] with an annealing temperature of 5 s at 54 °C during the amplification reaction. For each individual two sequences were obtained, from forward and reverse primers (see primer sequences in Table 1). Both sequences were assembled using SeqMan II from the Lasergene program (DNASTAR) to get one final sequence per individual. All sequences were then aligned with Clustal W, in BioEdit v.7.2 [52]. The sequence length of the total alignment for each of the four analysed genomic regions is given in Table 1 with the 5’UTR, exonic and intronic regions assigned by comparison with the orthologous sequences of *Drosophila pseudoobscura* retrieved from Flybase and visual inspection of the resulting expected proteins. The number of males analysed per locality and inversion are presented for each gene fragment in Table 2. Our sampling strategy targeted a comparable number of males to be analysed for each population and arrangement, and as such the inversion frequencies in our samples do not match natural inversion frequencies as seen in Fig. 1.

**Table 2:** Nucleotide variation for each gene and arrangement across all localities

*PhKgamma*					*Ubc-E2H*				*Hfw*		*Nipsnap*	
	Mal	Ras	Dij	Gro	Mal	Ras	Dij	Gro	Mal	Gro	Mal	Gro
A_ST_												
*n*	9	9	10	10	9	10	10	8	7	9	9	10
*h*	6	9	10	8	9	10	10	8	7	9	9	10
*S*	9	13	14	9	12	15	16	11	34	24	27	35
π	0.0031	0.0057	0.0055	0.0031	0.0038	0.0040	0.0047	0.0031	0.0105	0.0070	0.0084	0.0097
π_sil_	0.0034	0.0052	0.0042	0.0020	0.0038	0.0058	0.0055	0.0034	0.0142	0.0103	0.0090	0.0102
θ_sil_	0.0044	0.0055	0.0048	0.0027	0.0043	0.0071	0.0073	0.0044	0.0175	0.0120	0.0099	0.0124
K_sil_	0.1912	0.1926	0.1910	0.1887	0.3865	0.3780	0.3914	0.3799	0.2548	0.2474	0.4104	0.4138
A_2_												
*n*	10	8	9	10	10	9	10	10	10	10	10	10
*h*	5	3	3	5	8	9	8	6	10	10	9	10
*S*	5	8	2	4	12	11	8	6	19	18	24	22
π	0.0014	0.0025	0.0008	0.0012	0.0028	0.0030	0.0020	0.0015	0.0053	0.0052	0.0068	0.0056
π_sil_	0.0012	0.0026	0.0009	0.0008	0.0033	0.0028	0.0011	0.0014	0.0085	0.0095	0.0070	0.0057
θ_sil_	0.0021	0.0040	0.0011	0.0011	0.0050	0.0042	0.0020	0.0020	0.0102	0.0108	0.0084	0.0076
K_sil_	0.1988	0.1981	0.1999	0.1988	0.3728	0.3728	0.3714	0.3716	0.2469	0.2483	0.4144	0.4177

Note: *n* - sample size; *h* - number of haplotypes; *S* - number of polymorphic sites; π - nucleotide diversity

π_sil_ - nucleotide diversity in silent sites (synonymous and non-coding positions); θ_sil_ - heterozygosity in silent sites

K_sil_ - divergence per silent site using *D. pseudoobscura* as outgroup

### Data analysis and statistical methods

Several standard parameters for each locality and arrangement were estimated using DnaSP v5 [53]: the number of haplotypes (*h*), number of polymorphic sites (*S*), the nucleotide diversity (π), nucleotide diversity in synonymous sites and non-coding positions (π_sil_), heterozygosity in silent sites (ϴ_sil_) and divergence per silent site (K_sil_) using *D. pseudoobscura* as outgroup [54], with the Jukes-Cantor correction. Comparisons of the diversity values across localities and arrangements were performed with the Wilcoxon matched pairs test using Statistica v10. Genetic differentiation between or within arrangements was assessed using F_ST_ [55] and D_xy_ [56] as well as significant levels for Snn [57] were obtained with 10,000 replicates. False Discovery rate - FDR - correction [58] was applied to account for multiple comparisons in the differentiation analyses. Analyses were performed excluding gaps. For a visual representation of the genetic differences between arrangements and populations in the *PhKgamma* and *Ubc-E2H* gene regions, pairwise F_ST_ values were used to compute a Principal Coordinate Analysis (pcoa in R package ape version 3.5 (http://ape-package.ird.fr/). A locus-by-locus analysis of Molecular variance (AMOVA) was performed on Arlequin v3.5.2 [59] to test for genetic differentiation (F_CT_) between arrangements at each specific polymorphic site. A hierarchical structure was considered with two groups corresponding to the two arrangements (A_2_ vs A_ST_), with the different localities and individuals sampled within these groups.

Gene conversion tracts (GCTs) were detected using the algorithm defined by [60]. Linkage disequilibrium (LD) between pairs of informative sites was assessed and the statistical significance analysed with a chi-square test after Bonferroni correction. These analyses were performed for each arrangement and gene region, as well as for the concatenated dataset. The overall linkage disequilibrium statistic *ZnS* was estimated for informative sites. LD was also estimated between polymorphic informative sites through *r*^*2*^ estimates. Deviations from neutrality were tested using Tajima’s *D* [61] for each chromosomal arrangement and locality. Population size changes were also determined using R^2^ [62]. Statistical significance was obtained by coalescent simulations (5000 replicates) considering free recombination, as it seems most suitable when analysing *D. subobscura* genes whether located inside or outside chromosomal arrangements (see [33]). All neutrality tests were performed after removing recombinant individuals (with indication of GCTs). The DnaSP v5 program [53] was used to perform the GCTs and linkage disequilibrium analyses as well as neutrality tests.

## Results

### Nucleotide variability and genetic differentiation

**Fig. 3:**
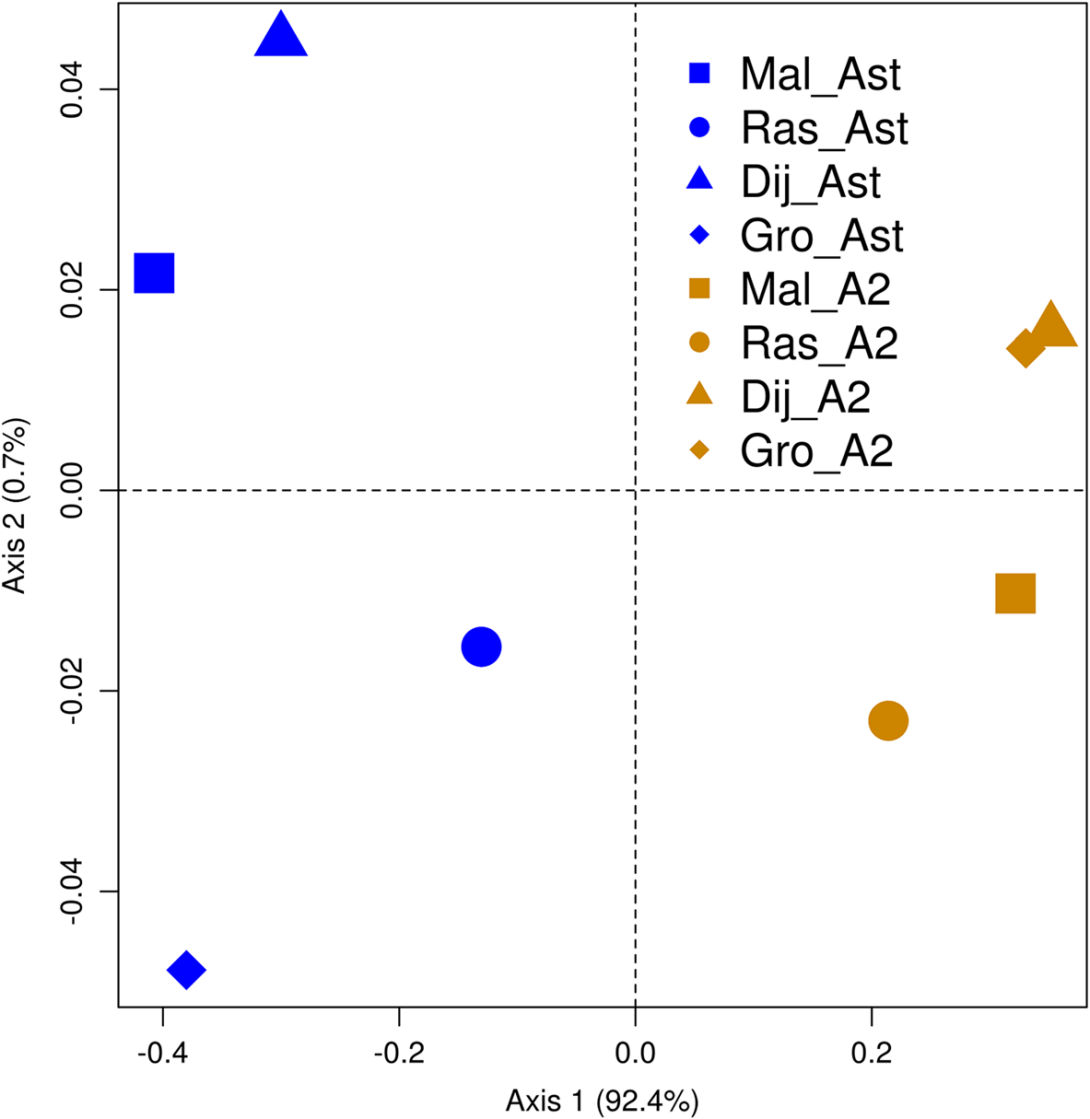
Principal Coordinate analysis based on genetic differentiation (F_ST_) among arrangements and localities for the *PhKgamma* gene. A_ST_ groups are represented in blue and A_2_ in orange; different localities are indicated by different symbols

**Fig. 4:**
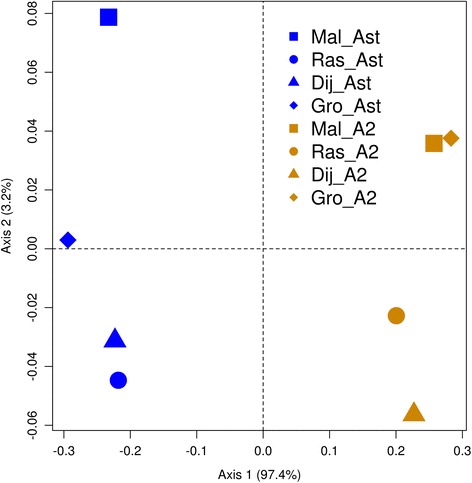
Principal Coordinate analysis based on genetic differentiation (F_ST_) among arrangements and localities for the *Ubc-E2H* gene. A_ST_ groups are represented in blue and A_2_ in orange; different localities are indicated by different symbols

For the two arrangements (A_ST_ and A_2_), we analysed the four genomic regions in the two most geographically distant localities (Málaga and Groningen), and two gene regions for the two central localities (Rasquera and Dijon) (Table 2). The two gene fragments being analysed in all localities were *PhKgamma* and *Ubc-E2H* as they showed genetic differentiation between arrangements (see Figs. 3 and 4 and Additional file 2). The genetic variation in those regions is likely to be affected by inversions to a greater extent and thus could possibly present clinal variation.

Diversity values were in general quite similar between localities for the same gene and chromosomal arrangement (see Additional file 3). Higher nucleotide diversity was found in A_ST_ than A_2_ arrangements at all gene regions (Table 2 and Additional file 3), with significant differences when including information from all regions and localities for all diversity measures (Wilcoxon matched pairs test; Z = 3.06, *P* = 0.002 for π and π_sil_; Z = 2.93, *P* = 0.003 for *S*; Z = 2.98, *P* = 0.003 for Ɵ_sil_). No differences in the divergence per silent site values (K_sil_) relative to *D. pseudoobscura* were found between the two arrangements (Wilcoxon matched pairs test; Z = 0.24, *P* = 0.814). Higher nucleotide diversity was found for *Hfw* and *Nipsnap* (Table 2 and Additional file 3), which might possibly indicate a higher substitution rate in these two more distally located regions.

High significant genetic differentiation between the A_ST_ and A_2_ arrangements was detected within each locality and also between localities for *Ubc-E2H* and *PhKgamma* (see Figs. 3 and 4 and Additional file 2) and also the concatenated dataset of all genes (see Additional file 4). On the other hand, *Hfw* and *Nipsnap* did not show significant differentiation between arrangements whether from the same or different localities (see Additional file 4). In general, no significant differentiation was observed when analysing groups of individuals carrying the same arrangement across localities with the exception of the comparison of A_ST_ individuals from Málaga and Rasquera in *PhKgamma* (see Additional file 2 and Additional file 4).

As low levels of within-population variation – as is the case in *PhKgamma* and *Ubc-E2H* – can inflate F_ST_ estimates [63, 64] we have used D_xy_, which is not affected by such low variation. The results for each gene region were qualitatively similar to those reported above for F_ST_, with consistently higher between-arrangement than within-arrangement differentiation for *PhKgamma* and *Ubc-E2H*, although the magnitude of such differences was lower than that obtained with F_ST_ estimates (Additional file 2). However, both statistics are highly correlated as shown by a Mantel test for *PhKgamma* (r^2^ = 0.754, *P* = 0.003) and *Ubc-E2H* (*r* = 0.741, *P* = 0.020).

**Fig. 5:**
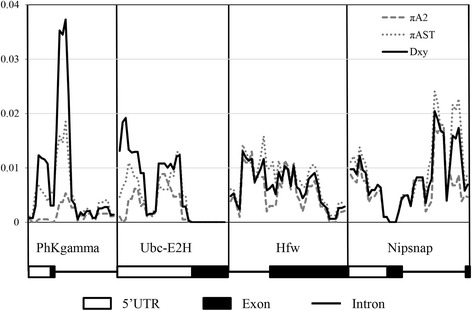
Genetic diversity (π) within each arrangement and D_xy_ between arrangements along all gene regions. D_xy_ – black line; π A_ST_ – dashed line; π A_2_ – dotted line. 5’UTR, intronic and exonic regions are discriminated (see also Table 1). The four gene regions are ordered following the A_ST_ arrangement (see Fig. 2)

Analysis of genetic differentiation, done separately in coding (exon) and non-coding (non-exon) regions for the four genes concatenated data set for Málaga and Groningen (the two geographically distant localities), showed that the significant differentiation between groups of individuals carrying different arrangements was due solely to variation in non-coding regions (see Additional file 5). Interestingly, these analyses indicated low but significant differentiation for A_ST_ individuals from different locations in non-coding regions, but not for A_2_ individuals. Considering each gene region, we found high differentiation between arrangements in upstream regions for *Ubc-E2H* and both upstream and intronic regions for *PhKgamma* while no relevant differentiation was observed for the other two genes in any region analysed (Fig. 5, see also Additional file 5). Curiously, in the intronic region of *PhKgamma* significant genetic differentiation between A_ST_ individuals from different localities was observed (see Additional file 5). A locus-by-locus analysis of Molecular variance (AMOVA) was performed to test the genetic differentiation (F_CT_) between arrangements at each specific polymorphic site using a hierarchical structure (see Methods). Again, differentiation between arrangements was observed exclusively in non-coding regions (see Additional file 6).

On average F_ST_ values between arrangements found in the same locality or in different localities showed similar values in both *PhKgamma* and *Ubc-E2H* and the concatenated dataset (see Additional file 2 and Additional file 4; see also Figs. 3 and 4). In these regions, differentiation between arrangements was similar across localities with the exception of Rasquera for *PhKgamma* (see Additional file 7). However, this exception was due to the recombinant individuals from Rasquera (four individuals presenting gene conversion tracks, see below).

Pairwise genetic distances, based on the concatenated dataset of *PhKgamma* and *Ubc-E2H*, were correlated with their geographic distance with Mantel tests. No significant association between genetic and geographic distances was found for individuals carrying A_2_ (r^2^ = 0.004, *P* = 0.462), or A_ST_ (r^2^ = 0.017, *P* = 0.284). Similar results are also obtained for the two genes separately (see Figs. 3 and 4). In agreement with this, no particular haplotype in any arrangement showed consistent indications of directional changes in frequency with latitude.

Gene conversion tracts (GCT) were detected only in *PhKgamma* and in four individuals, all of them from Rasquera. When removing individuals presenting GCTs in the *PhKgamma* gene, four fixed differences were detected between arrangements (position 205 in the 5’UTR, and positions 358–360 in the intron). For *Ubc-E2H*, one fixed difference was found between the two arrangements (position 60 in the 5’UTR). No fixed differences were found in the other two genes.

### Linkage disequilibrium

In general, levels of linkage disequilibrium (LD) were low in all gene regions and arrangements analysed (see Additional file 8). Using the concatenated dataset of all four genes and considering both arrangements, LD between pairs of polymorphic sites (R^2^) showed only 35 significant comparisons out of a total of 2415 (*P* < 0.001, chi-square with Bonferroni correction). Importantly, the majority of the significant LD comparisons were observed between sites from the same gene region, indicating short-range LD (see also Additional file 9). In fact, only 12 of the 35 significant comparisons corresponded to LD between distinct gene regions - 6 of them between *PhKgamma* and *Ubc-E2H* (namely between positions 358-360 bp from *PhKgamma* and position 60 bp from *Ubc-E2H*, see below). In the analysis including only the individuals carrying the A_2_ inversion just 3 out of a total of 496 comparisons indicated significant LD after Bonferroni correction (*P* < 0.001, chi-square with Bonferroni correction). However, in these three cases, the significant LD comparisons were always between sites within the same gene region (see Additional file 9). No significant comparisons were found between polymorphic sites for A_ST_ individuals. Importantly, these results show that within-arrangement LD was not observed across populations. However, fixed associations (complete LD) were found between arrangements and sites in *PhKgamma* (positions 205, 358–360 of the total alignment with *D. pseudoobscura*; *P* < 0.001, chi-square with Bonferroni correction) and also in *Ubc-E2H* (position 60; *P* < 0.001, chi-square with Bonferroni correction). Interestingly, all positions in *PhKgamma* are also in complete LD among themselves (*P* < 0.001, chi-square with Bonferroni correction). All these sites are located in non-coding/intronic regions. The fixed position in the *Ubc-E2H* gene is located 547 bp upstream from the first exon. The fixed positions at 358–360 bp in the *PhKgamma* gene are located in an intronic region just 26 bp downstream of the first exon. Comparison against *D. pseudoobscura* orthologous gene indicates more similarity to A_ST_ sequences. The fixed position between arrangements at 205 bp is within the 5’UTR region 36 bp upstream of the start coding site of *PhKgamma*, and possibly within the core promoter region, despite the absence of characteristic sequence motifs such as TATA box and the TFIIB recognition element [65].

### Neutrality tests

All Tajima’s *D* values were negative for each gene, arrangement and locality, and also for all locations combined (see Additional file 10). Tajima’s *D* values were generally more negative in the A_2_ arrangement. R_2_ values also indicated stronger deviations from neutrality in the A_2_ arrangement (see Additional file 10). The general scenario points to an excess of low frequency variants, consistent with a population expansion scenario, particularly in the A_2_ arrangement.

## Discussion

A_ST_ and A_2_ are respectively a cold and a warm-adapted arrangement: while A_ST_ shows increased frequencies towards higher, colder latitudes, the opposite pattern is found for A_2_ [32, 44], with both gene flow and local adaptation likely implicated [7]. In this study we analysed patterns of genetic variation associated with these arrangements and searched for patterns of geographical selection at the genetic level in candidate genes for thermal adaptation [40], located within or near these inverted regions.

Our study shows that these arrangements shape the patterns of genetic variation likely by reducing recombination between certain regions within chromosomes. In fact, the two proximally located regions in A_ST_ (*PhKgamma* and *Ubc-E2H*) presented significant differentiation between arrangements particularly in non-coding regions, regardless of the arrangement’s geographical origin. Several molecular studies in *Drosophila* have documented significant genetic differentiation between chromosomes carrying different arrangements, particularly within inverted regions [22, 23, 25, 29, 33, 66] and also specifically near inversion breakpoints (e.g. [14, 21, 23]). The differentiation between arrangements that we observe could result from locally adapted alleles protected within the inversions which would be in agreement with the expectations of both the coadaptation and local adaptation hypotheses [17, 18]. Furthermore, the fact that these differences are observed in 5’UTR and intronic regions might indicate that regulatory regions could be mostly the targets of selection (see [67]). In addition, the low differentiation between arrangements at coding regions - and the low variability observed - might be an effect of purifying selection acting in these regions. However, interpreting these patterns as being due to local adaptation is not straightforward, as neutral processes might also be involved. Non-adaptive associations between the inversion and neutral genetic variants generated during the origin and/or initial spread of the inversion could lead to longstanding differentiation and linkage disequilibrium [68], particularly near breakpoints where recombination is expectedly very low [19, 69]. This signal is more likely to be maintained when population size is high and the inversion is young – see [19]. In our case, despite the high effective size of the species [70] the age of A_2_ - around 160.000 years [23], makes this expectation less likely. Moreover, the fact that microsatellite loci located in the same (proximal) regions as *PhKgamma* and *Ubc-E2H* did not show similar patterns [32] - namely the observed depletion in genetic variation and increase in differentiation between arrangements - further suggests that selection might be involved in the regions analysed here.

In the specific context of the A_ST_/A_2_ complex, Nóbrega et al. [23] also found that the extent of genetic exchange between arrangements was limited, and not sufficient to homogenize nucleotide variation in the centre of A_2_. In our study, we show that the reduction of recombination is not homogeneous in regions around the vicinity of the breakpoints of the A_ST_/A_2_ complex, with significant genetic differentiation and linkage disequilibrium between inversions occurring only in the proximal region of A_ST_. Interestingly, linkage disequilibrium occurred in one region (*PhKgamma*) located outside the inversion, although near the breakpoint. Other studies have suggested that suppressed recombination can extent to 2.5 Mb or more outside inversion breakpoints [25, 71]. Functional analyses might elucidate if the non-coding variation regions found in *PhKgamma* and *Ubc-E2H* is in fact adaptive.

We did not find evidence for geographical (i.e. climatic) selection within arrangements, as very low within-arrangement genetic differentiation was detected across localities. It is possible that the differences in gene expression described previously for these genomic regions between thermal regimes [40] were controlled by other genes/sequences besides the upstream and coding regions analysed here. Importantly, the differential expression [40] was observed at the population level -between populations evolving under cold (13 °C) vs warm (22 °C) regimes - and not addressed at the karyotype level. It is possible that the gene expression in a particular karyotype does not present clinal variation, with differential gene expression occurring between distinct karyotypes. Most interestingly, the expression of *PhKgamma* and *Nipsnap* have shown to be positively associated to latitude in semi natural populations of *D. subobscura* from a similar geographic gradient, but not when the same populations were maintained in a common garden [72]. Thus, although evidence is not conclusive, these gene regions could be involved in local adaptation to temperature.

Other studies in *D. subobscura* found very limited evidence for geographical molecular variation within inversions. Pegueroles et al. [33] also analysed candidate genes for thermal adaptation located in the O chromosome of *D. subobscura* and found no significant differentiation within each arrangement for individuals sampled in Barcelona (Spain) and Mount Parnes (Greece). Evidence for similar patterns of generally low within-arrangement differentiation was also described in other *D. subobscura* studies [73, 74] as well as studies in *D. melanogaster* ([31], but see [75]) and *D. pseudoobscura* [20]. However, recent evidence in *D. melanogaster* indicates several instances of genetic clinal variation in regions linked with inversions particularly associated with the *In(3R)Payne* [6, 34], perhaps due to the higher resolution power of these later studies in terms of the genomic scan performed and number of sampling sites studied.

Within-arrangement differentiation across localities would be expected if different epistatic interactions evolved (and eventually got fixed) in different localities due to adaptation to the specific local conditions as proposed by the Dobzhansky’s coadaptation model [17]. The lack of within-arrangement differentiation of the selected genes across populations suggests that population-specific epistatic interactions are not occurring in the candidate genes for thermal adaptation that we sampled. It is of course possible that other loci within the inversion might present such varying epistatic interactions in the different localities. Some studies have reported evidence of epistasis based on patterns of low LD between neighbouring sites and high LD between distant sites within the inversion [20, 31]. Recently, indications of epistatic selection have also been obtained from observations of regions with significant genetic differentiation outside arrangements presenting strong linkage disequilibrium with the arrangements. In *Atpα* gene, another candidate gene for thermal adaptation, Pegueroles et al. [30] found strong genetic differentiation associated to the analysed arrangements of the O chromosome despite this gene being located outside them. Higher resolution genomic scans of adaptive variation as well as functional analyses are needed to conclusively assess the presence of relevant epistatic interactions and if these differ across populations.

## Conclusions

Our genetic analysis of candidate loci for thermal adaptation [40] across geographically distinct *D. subobscura* localities of the European cline found very low within-arrangement differentiation across localities as well as no clinal patterns of genetic variation. This indicates that gene flow across the European continent has homogenized the genetic content of these potentially selected regions at a wide geographical scale. Given the strong effect of gene flow in this species, it is possible that considerable within-arrangement differentiation at a geographical scale is only maintained at relatively few loci subjected to stronger selective pressures in the different environments. In this scenario, one can argue that restricted gene flow cannot explain the observed inversion frequency clines and that local adaptation is maintaining the clinal variation in this species, counteracting the homogenizing effects of the high gene flow. We also found an important effect of the A_ST_/A_2_ complex in shaping genetic variation and preventing relevant gene exchange in different genomic regions, although its impact was not uniform across the different regions analysed. More generally, our study adds to a body of evidence showing that inversions act as barriers to recombination even outside inversions and can potentially have a role in the spread of favourable genic combinations and consequent adaptation to local habitats.

## Additional files

Additional file 1: New hybridization location for *Nipsnap* gene. (PDF 108 kb)

Additional file 2: Measures of genetic differentiation within and between arrangements for *PhKgamma* and *Ubc-E2H*. A) Genetic differentiation (F_ST_) between all localities and arrangements in *PhKgamma* (below diagonal) and *Ubc-E2H* (above diagonal). B) Genetic differentiation (D_xy_) between all localities and arrangements in *PhKgamma* (below diagonal) and *Ubc-E2H* (above diagonal). (XLSX 12 kb)

Additional file 3: Nucleotide diversity (π) for all genes, arrangements and localities. (PDF 178 kb)

Additional file 4: Genetic differentiation (F_ST_) between Malaga and Groningen in *Hfw*, *Nipsnap* and the concatenated dataset (four loci). (XLSX 10 kb)

Additional file 5: Genetic differentiation (F_ST_) for coding and non-coding regions in the concatenated dataset for all 4 genes (A) and for coding, intronic and 5’ UTR regions for *PhKgamma* (B). (XLSX 13 kb)

Additional file 6: Genetic differentiation between arrangements (F_CT_) for all genes. 5’UTR, intronic and exonic regions are discriminated (see also Table 1). The four gene regions depicted are not contiguous. (PDF 238 kb)

Additional file 7: Genetic differentiation between arrangements for each locality in *PhKgamma* (A) and *Ubc-E2H* (B). (PDF 344 kb)

Additional file 8: Linkage Disequilibrium parameters for all regions and the concatenated dataset. (XLSX 10 kb)

Additional file 9: Linkage disequilibrium (LD) between pairs of polymorphic sites (R^2^) against the nucleotide distance between compared sites. Significant comparisons after Bonferroni correction are presented in red (see Methods for additional details). A) LD between arrangements; B) LD within A_ST_; C) LD within A_2_. (PDF 248 kb)

Additional file 10: Neutrality tests and test of population expansion for each arrangement and gene. (XLSX 36 kb)

## Abbreviations

AMOVA: Analysis of Molecular Variance
FDR: False discovery rate
GCT: Gene conversion track
LD: Linkage disequilibrium
PCR: Polymerase chain reaction
UTR: Untranslated region
