# Supplementary figures and images for: Patterns of geographic variation of thermal adapted candidate genes in Drosophila subobscura sex chromosome arrangements
Source: BMC Evol Biol. 2018 Apr 24;18:60. doi: 10.1186/s12862-018-1178-1 (PMC5921438; doi:10.1186/s12862-018-1178-1)

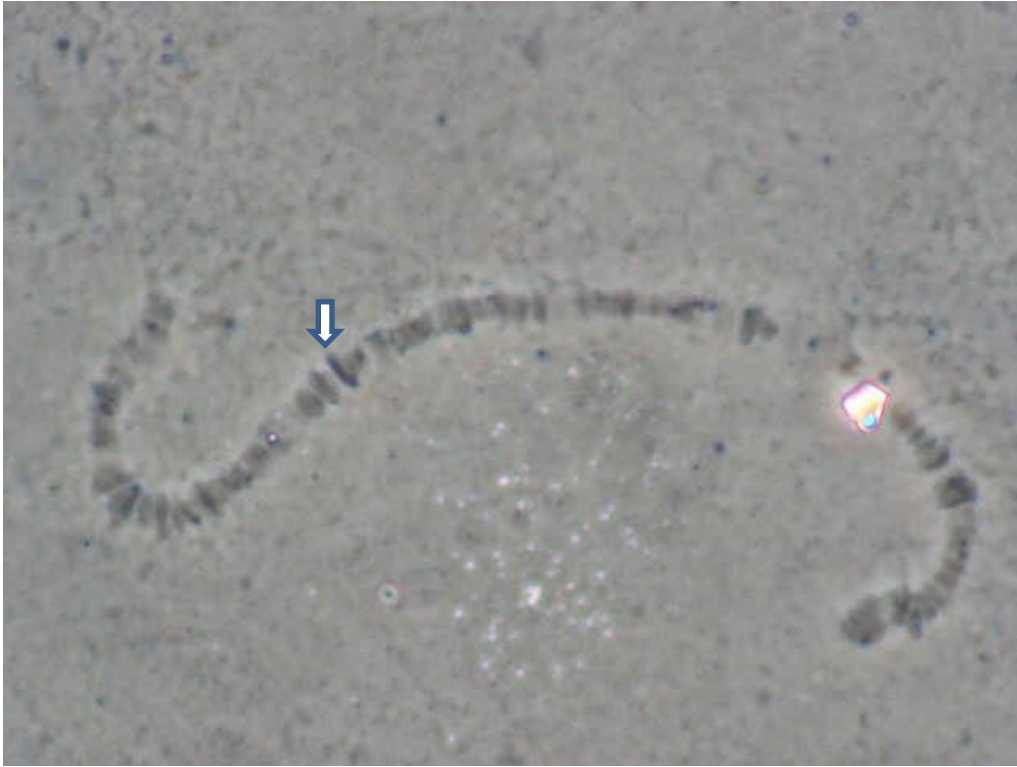

Additional file 1 - New hybridization location for Nipsnap.

Supplement: Supplementary file 1 — New hybridization location for Nipsnap gene. (PDF 108 kb) [file 12862_2018_1178_MOESM1_ESM.pdf]

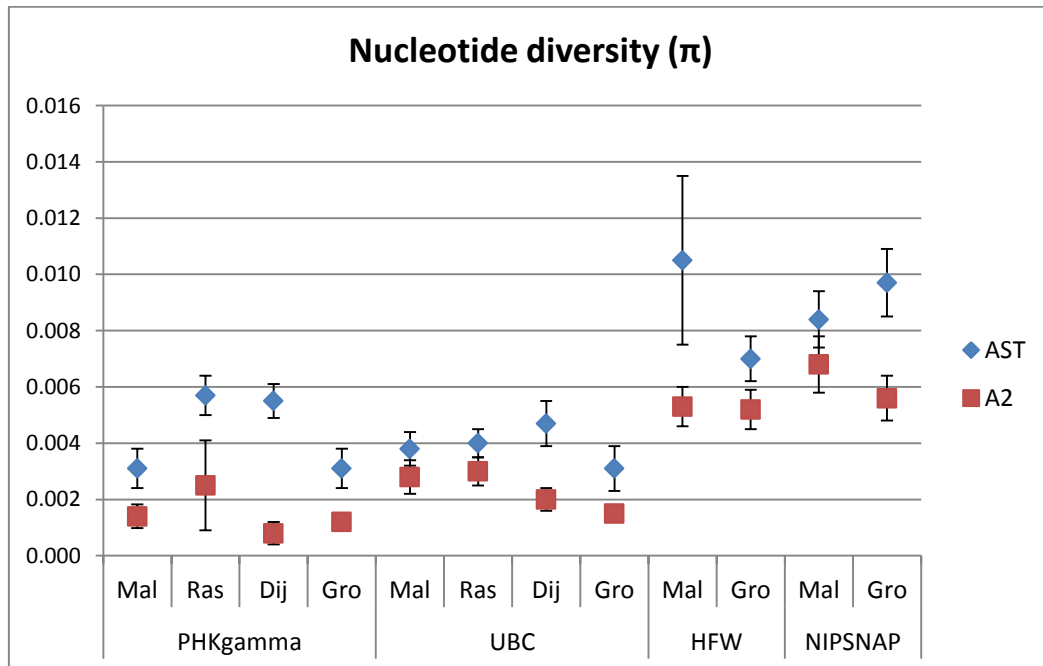

Additional file 3 – Nucleotide diversity for all genes, arrangements and localities.

Supplement: Supplementary file 3 — Nucleotide diversity (π) for all genes, arrangements and localities. (PDF 178 kb) [file 12862_2018_1178_MOESM3_ESM.pdf]
