# Supplementary material for: Patterns of geographic variation of thermal adapted candidate genes in Drosophila subobscura sex chromosome arrangements
Source: BMC Evol Biol. 2018 Apr 24;18:60. doi: 10.1186/s12862-018-1178-1 (PMC5921438; doi:10.1186/s12862-018-1178-1)
Supplement: Supplementary file 6 — Genetic differentiation between arrangements (FCT) for all genes. 5’UTR, intronic and exonic regions are discriminated (see also Table 1). The four gene regions depicted are not contiguous. (PDF 238 kb) [file 12862_2018_1178_MOESM6_ESM.pdf]

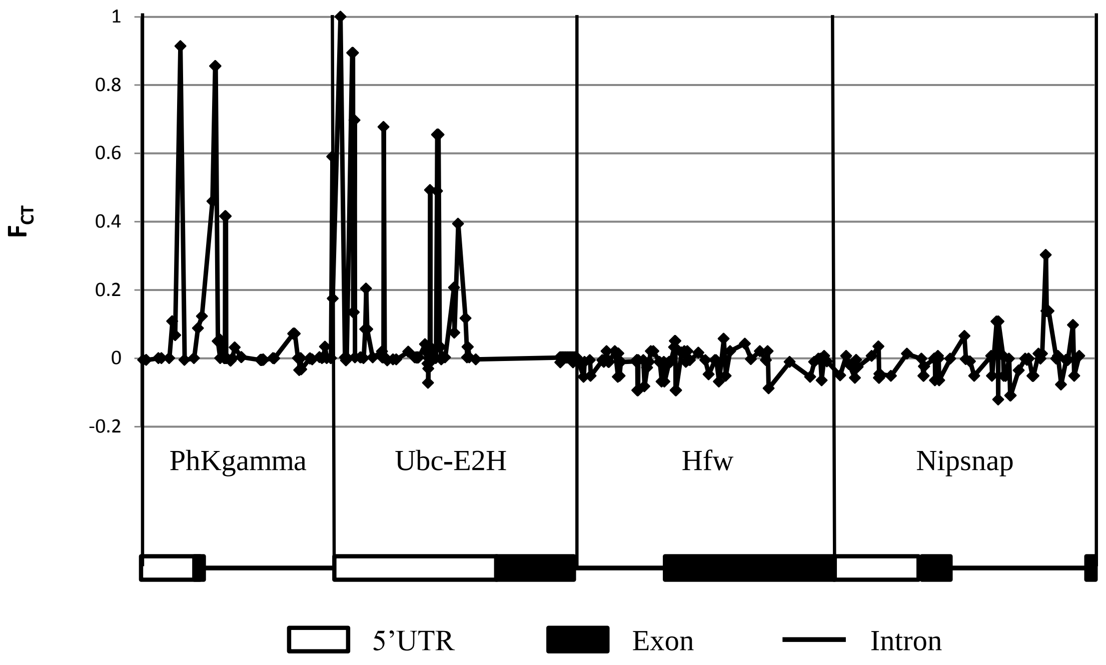

Additional file 6 - Genetic differentiation between arrangements (F_{CT}) for all genes. 5’UTR, intronic and exonic regions are discriminated (see also Table 1). The four gene regions depicted are not contiguous.
