# Supplementary material for: Patterns of geographic variation of thermal adapted candidate genes in Drosophila subobscura sex chromosome arrangements
Source: BMC Evol Biol. 2018 Apr 24;18:60. doi: 10.1186/s12862-018-1178-1 (PMC5921438; doi:10.1186/s12862-018-1178-1)
Supplement: Supplementary file 7 — Genetic differentiation between arrangements for each locality in PhKgamma (A) and Ubc-E2H (B). (PDF 344 kb) [file 12862_2018_1178_MOESM7_ESM.pdf]

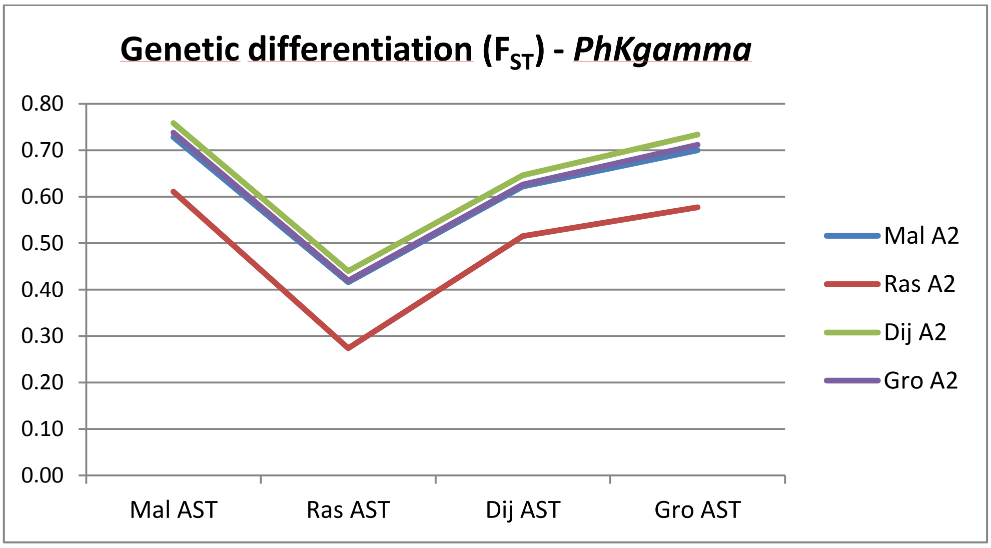

A) Genetic differentiation in *PhKgamma*

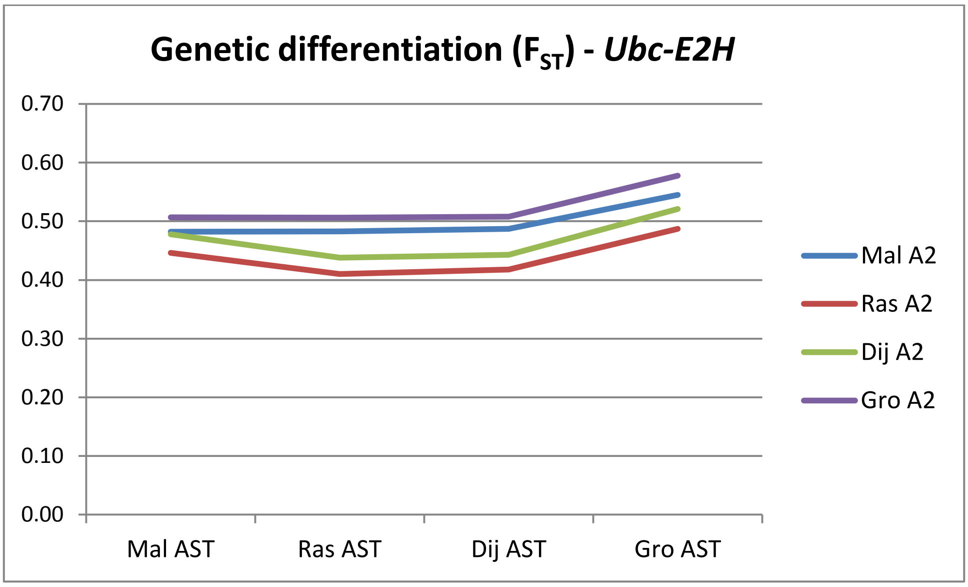

B) Genetic differentiation in *Ubc-E2H*

Additional file 7 – Genetic differentiation between arrangements for each locality in *PhKgamma* (A) and *Ubc-E2H* (B).
