# Supplementary material for: Patterns of geographic variation of thermal adapted candidate genes in Drosophila subobscura sex chromosome arrangements
Source: BMC Evol Biol. 2018 Apr 24;18:60. doi: 10.1186/s12862-018-1178-1 (PMC5921438; doi:10.1186/s12862-018-1178-1)
Supplement: Supplementary file 9 — Linkage disequilibrium (LD) between pairs of polymorphic sites (R2) against the nucleotide distance between compared sites. Significant comparisons after Bonferroni correction are presented in red (see Methods for additional details). A) LD between arrangements; B) LD within AST; C) LD within A2. (PDF 248 kb) [file 12862_2018_1178_MOESM9_ESM.pdf]

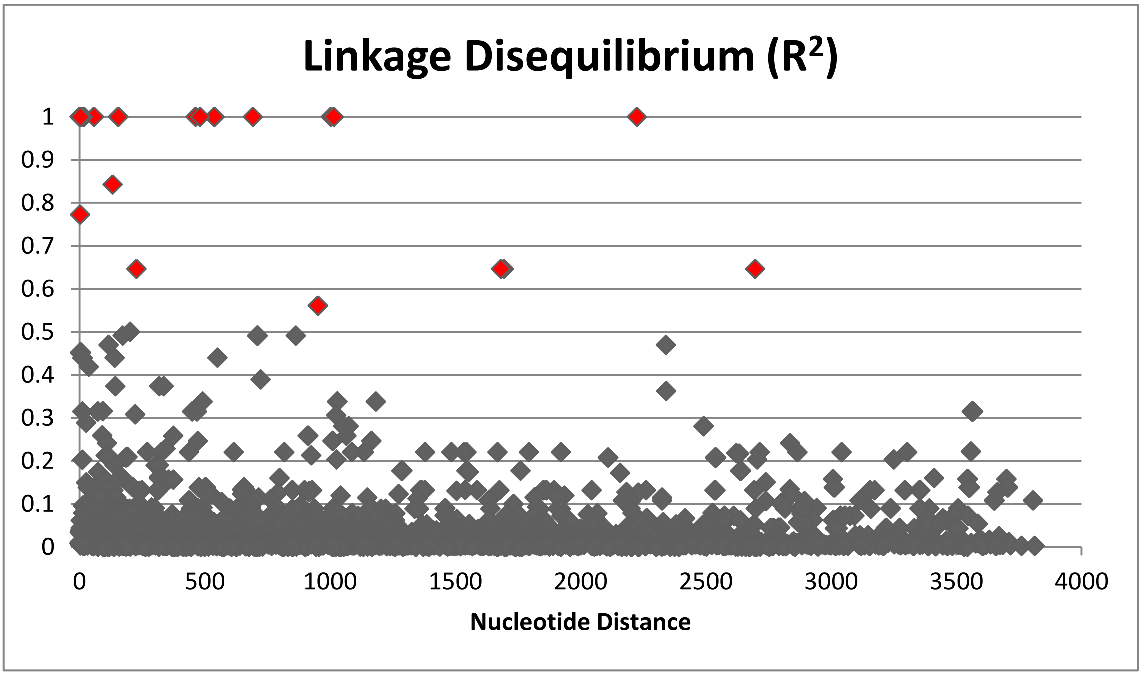


A) LD between arrangements

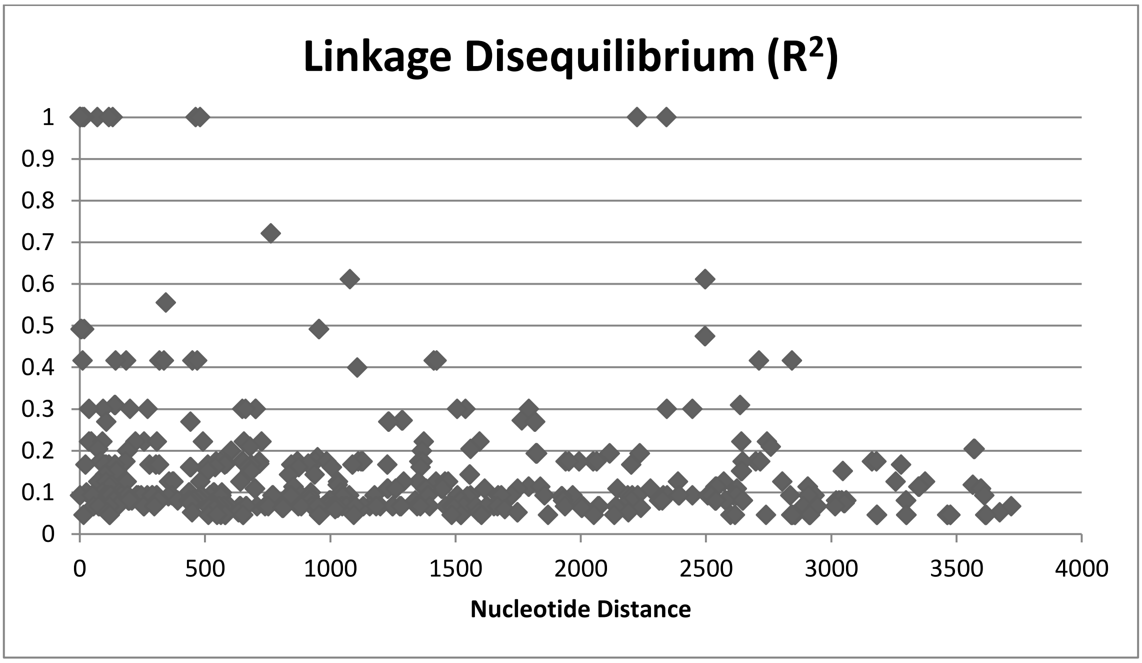


B) LD within $A_{ST}$

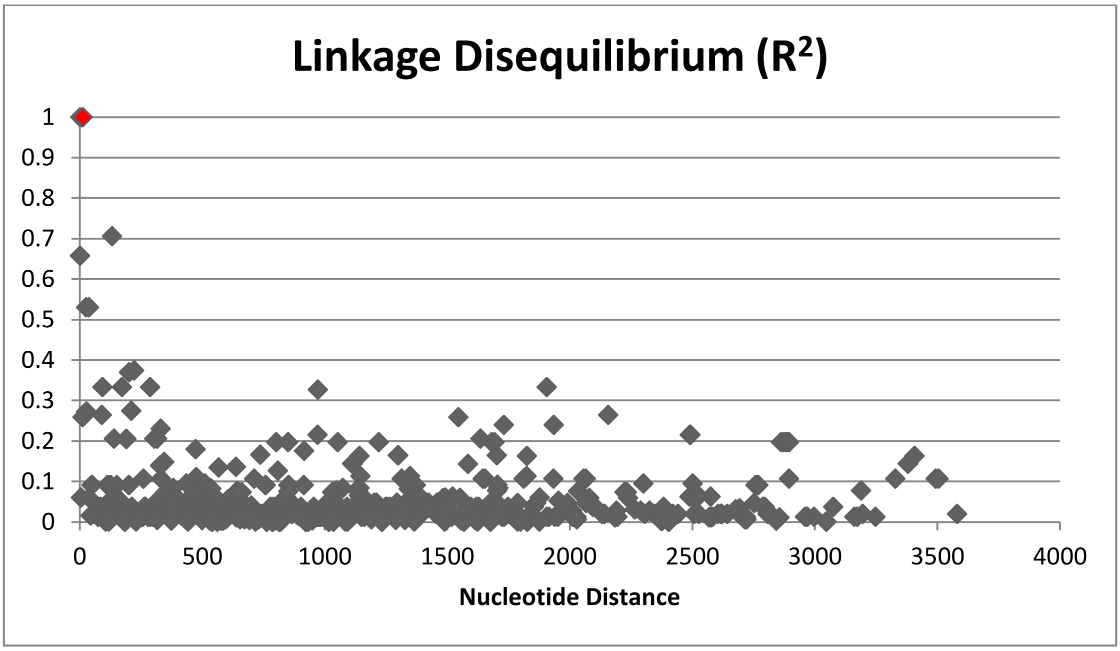


C) LD within $A_2$

Additional file 9 – Linkage disequilibrium (LD) between pairs of polymorphic sites ($R^2$) against the nucleotide distance between compared sites. Significant comparisons after Bonferroni correction are presented in red (see Methods for additional details). A) LD between arrangements; B) LD within $A_{ST}$; C) LD within $A_2$.
